# Offline Digital Education for Postregistration Health Professions: Systematic Review and Meta-Analysis by the Digital Health Education Collaboration

**DOI:** 10.2196/12968

**Published:** 2019-04-24

**Authors:** Pawel Posadzki, Malgorzata M Bala, Bhone Myint Kyaw, Monika Semwal, Ushashree Divakar, Magdalena Koperny, Agnieszka Sliwka, Josip Car

**Affiliations:** 1 Centre for Population Health Sciences (CePHaS) Lee Kong Chian School of Medicine Nanyang Technological University Singapore Singapore Singapore; 2 Chair of Epidemiology and Preventive Medicine Department of Hygiene and Dietetics Jagiellonian University Medical College Krakow Poland; 3 Family Medicine and Primary Care Lee Kong Chian School of Medicine Nanyang Technological University Singapore Singapore; 4 Province Sanitary-Epidemiological Station of Lesser Poland Public Health and Health Promotion Department Krakow Poland; 5 Institute of Physiotherapy, Faculty of Health Sciences Jagiellonian University Medical College Krakow Poland

**Keywords:** randomized controlled trial, systematic review, medical education

## Abstract

**Background:**

The shortage and disproportionate distribution of health care workers worldwide is further aggravated by the inadequacy of training programs, difficulties in implementing conventional curricula, deficiencies in learning infrastructure, or a lack of essential equipment. Offline digital education has the potential to improve the quality of health professions education.

**Objective:**

The primary objective of this systematic review was to evaluate the effectiveness of offline digital education compared with various controls in improving learners’ knowledge, skills, attitudes, satisfaction, and patient-related outcomes. The secondary objectives were (1) to assess the cost-effectiveness of the interventions and (2) to assess adverse effects of the interventions on patients and learners.

**Methods:**

We searched 7 electronic databases and 2 trial registries for randomized controlled trials published between January 1990 and August 2017. We used Cochrane systematic review methods.

**Results:**

A total of 27 trials involving 4618 individuals were included in this systematic review. Meta-analyses found that compared with no intervention, offline digital education (CD-ROM) may increase knowledge in nurses (standardized mean difference [SMD]=1.88; 95% CI 1.14 to 2.62; participants=300; studies=3; *I^2^*=80%; low certainty evidence). A meta-analysis of 2 studies found that compared with no intervention, the effects of offline digital education (computer-assisted training [CAT]) on nurses and physical therapists’ knowledge were uncertain (SMD 0.55; 95% CI –0.39 to 1.50; participants=64; *I^2^*=71%; very low certainty evidence). A meta-analysis of 2 studies found that compared with traditional learning, a PowerPoint presentation may improve the knowledge of patient care personnel and pharmacists (SMD 0.76; 95% CI 0.29 to 1.23; participants=167; *I^2^*=54%; low certainty evidence). A meta-analysis of 4 studies found that compared with traditional training, the effects of computer-assisted training on skills in community (mental health) therapists, nurses, and pharmacists were uncertain (SMD 0.45; 95% CI –0.35 to 1.25; participants=229; *I*^2^=88%; very low certainty evidence). A meta-analysis of 4 studies found that compared with traditional training, offline digital education may have little effect or no difference on satisfaction scores in nurses and mental health therapists (SMD –0.07; 95% CI –0.42 to 0.28, participants=232; *I^2^*=41%; low certainty evidence). A total of 2 studies found that offline digital education may have little or no effect on patient-centered outcomes when compared with blended learning. For skills and attitudes, the results were mixed and inconclusive. None of the studies reported adverse or unintended effects of the interventions. Only 1 study reported costs of interventions. The risk of bias was predominantly unclear and the certainty of the evidence ranged from low to very low.

**Conclusions:**

There is some evidence to support the effectiveness of offline digital education in improving learners’ knowledge and insufficient quality and quantity evidence for the other outcomes. Future high-quality studies are needed to increase generalizability and inform use of this modality of education.

## Introduction

### Background

There is no health care system without health professionals. The health outcomes of people rely on well-educated nurses, pharmacists, dentists, and other allied health professionals [1]. Unfortunately, these professionals are in short supply and high demand [2,3]. Almost 1 billion people are negatively affected by the lack of access to adequately trained health professionals, suffering ill-health or dying [4,5]. In many low- and middle-income countries (LMICs), this situation is further aggravated by the difficulties in implementing traditional learning programs; deficiencies in health care systems and infrastructure; and lack of essential supplies, poor management, corruption, or low remuneration [6].

Digital education also known as e-learning is an umbrella term encompassing a broad spectrum of educational interventions characterized by their tools, technological contents, learning objectives or outcomes, pedagogical approaches, and delivery settings, which includes, but is not limited to, online and offline computer-based digital education, massive open online courses (MOOCs), mobile learning (mLearning), serious gaming and gamification, digital psychomotor skill trainers, virtual reality, or virtual patient scenarios [7]. Digital education aims to improve the quality of teaching by facilitating access to resources and services, as well as remote exchange of information and peer-to-peer collaboration [8]; it is also being increasingly recognized as one of the key strategic platforms to build strong education and training systems for health professionals worldwide [9]. The United Nations and the World Health Organization consider digital education as an effective means of addressing the educational needs among health professionals, especially in LMICs.

This review focused on offline digital education. This refers to the use of personal computers or laptops to assist in delivering stand-alone multimedia materials without the need for the internet or local area network connections [10]. The educational content can be delivered via videoconferences, emails, and audio-visual learning materials kept in either magnetic storage, for example, floppy disks, or optical storage, for example, CD-ROM, digital versatile disk, flash memory, multimedia cards, external hard disks, or downloaded from a networked connection, as long as the learning activities do not rely on this connection [11].

There are several potential benefits of offline digital education such as unrestrained knowledge transfer, enriched accessibility, and significance of health professions education [12]. Further benefits include flexibility and adaptability of educational content [13], so that learners can absorb curricula at a convenient pace, place, and time [14]. The interventions can also be used to deliver an interactive, an associative, and a perceptual learning experience by combining text, images, audio, and video via combined visual, auditory, and spatial components, further improving health professionals’ learning outcomes [15,16]. By doing so, offline digital education can potentially stimulate neurocognitive development (memory, thinking, and attention) by enhancing changes in the efficiency of chemical synaptic transmission between neurons, increasing specific neuronal connections and creating new patterns of neuronal connectivity and generating new neurons [17]. Finally, health professionals better equipped with knowledge, skills, or professional attitudes as a result of offline digital education might improve the quality of health care services provision, as well as the patient-centered and public health outcomes, and reduce the costs of health care.

### Objectives

This systematic review was one of a series of reviews evaluating the scope for implementation and the potential impact of a wide range of digital health education interventions for postregistration and preregistration health professionals. The objective of this systematic review was to evaluate the effectiveness of offline digital education compared with various controls in improving learners’ knowledge, skills, attitudes, satisfaction, and patient-centered outcomes.

## Methods

At the time of conducting and reporting the review, we used and adhered to the systematic review methods as recommended by the Cochrane Collaboration [18]. For a detailed description of the methodology, please refer to the study by Car et al [7].

### Search Strategy and Data Sources

We searched the following databases (from January 1990 to August 2017): MEDLINE (via Ovid), Excerpta Medica dataBASE (via Elsevier), Web of Science, Educational Resource Information Center (via Ovid), Cochrane Central Register of Controlled Trials, The Cochrane Library, PsycINFO (via Ovid), and the Cumulative Index to Nursing and Allied Health Literature (via EBSCO). The search strategy for MEDLINE is presented in Multimedia Appendix 1. We searched for papers in English but considered eligible studies in any language. We also searched 2 trial registries (EU Clinical Trials Register and ClinicalTrials.gov), screened reference lists of all included studies and pertinent systematic reviews, and contacted the relevant investigators for further information.

### Eligibility Criteria

Only randomized controlled trials (RCTs) and cluster RCTs (cRCTs) of postregistration health professionals except medical doctors—as they were covered in a separate review [19]—using either stand-alone or blended offline digital education with any type of controls (active or inactive) measuring knowledge, skills, attitudes, satisfaction, and patient-centered outcomes (as primary outcomes) as well as adverse effects or costs (as secondary outcomes) were eligible for inclusion in this review.

We excluded crossover trials, stepped wedge design, interrupted time series, controlled before and after studies, and studies of doctors (including medical diagnostics and treatment technologies) or medical students. Participants were not excluded on the basis of sociodemographic characteristics such as age, gender, ethnicity, or any other related characteristics.

### Data Selection, Extraction, and Management

The search results from the different electronic databases were combined in a single EndNote (X8.2) library, and duplicate records of the same reports were removed. In total, 2 reviewers independently screened titles and abstracts to identify studies that potentially meet the inclusion criteria. The full text versions of these articles were retrieved and read in full. Finally, 2 review authors independently assessed articles against the eligibility criteria, and 2 reviewers independently extracted the data for each of the included studies using a structured data extraction form and the Covidence Web-based software (Veritas Health Innovation, Melbourne, Australia). We extracted all relevant data on the characteristics of participants, intervention, comparator group, and outcome measures. For continuous data, we reported means and SDs and odds ratios (ORs) and its 95% CIs for dichotomous data. For studies with multiple arms, we compared the relevant intervention arm to the least active control arm, so that double counting of data does not occur. Any disagreements were resolved through discussion between the 2 authors and if no consensus was reached, a third author acted as an arbiter.

### Assessment of Risk of Bias

In total, 2 reviewers independently assessed the risk of bias of the included studies using the Cochrane Collaboration’s *Risk of Bias* tool [18]. Studies were assessed for risk of bias in the following domains: random sequence generation; allocation concealment; blinding of participants or personnel; blinding of outcome assessment; completeness of outcome data (attrition bias); selective outcome reporting (reporting bias); validity and reliability of outcome measures; baseline comparability; and consistency in intervention delivery. For cRCTs, we also assessed and reported the risk of bias associated with an additional domain: selective recruitment of cluster participants. Judgments concerning the risk of bias for each study fell under 3 categories: high, low, or unclear risk of bias.

### Data Synthesis

Data were synthesized using Review Manager version 5.3. In cases where studies were homogeneous enough (in terms of their population interventions, comparator groups, outcomes, and study designs) to make meaningful conclusions, we pooled them together in a meta-analysis using a random-effects model and presented results as standardized mean difference (SMD). We assessed heterogeneity through a visual inspection of the overlap of forest plots and by calculating the chi-square tests and *I*^*2*^ inconsistency statistics [18].

### Summary of Findings Tables

We prepared the Summary of Findings (SoF) tables to present the results for each of the primary outcomes. We converted results into absolute effects when possible and provided a source and rationale for each assumed risk cited in the table(s) when presented. A total of 2 authors (PP and MS) independently rated the overall quality of evidence as implemented and described in the GRADEprofiler (GRADEproGDT Web-based version) and Chapter 11 of the *Cochrane Handbook for Systematic Reviews of Interventions* [20]. We considered the following criteria to assess the quality of evidence: limitations of studies (risk of bias), inconsistency of results, indirectness of the evidence, imprecision and publication bias, and downgraded the quality where appropriate. This was done for all primary outcomes reported in the review.

## Results

**Figure 1:**
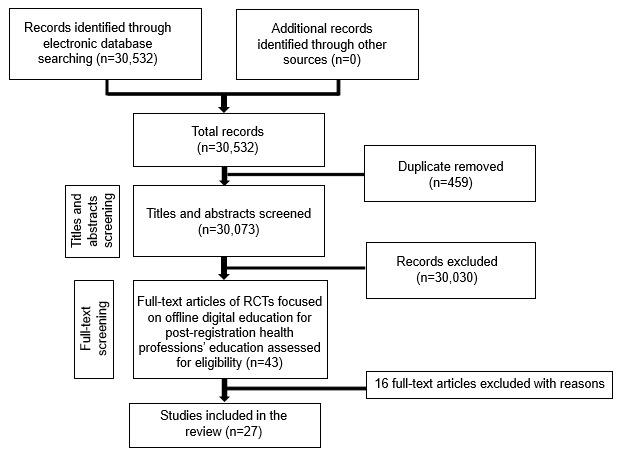
Study flow diagram. RCT: randomized controlled trial.

Our searches yielded a total of 30,532 citations; and 27 studies with 4,618 participants are included in Figure 1. For characteristics of excluded studies, please refer to Multimedia Appendix 2.

### Included Studies

Details of each trial are presented in Table 1 or Multimedia Appendix 3; a summary is given below. The included trials were published between 1991 and 2016, and originated from Brazil 3.7% (n=1), Hong Kong 3.7% (n=1), Iran 7.4% (n=2), Korea 3.7% (n=1), the Netherlands 11.1% (n=3), Norway 3.7% (n=1), Taiwan 11.1% (n=3), Turkey 3.7% (n=1), the United Kingdom 7.4% (n=2), and the United States 44.4% (n=12). A total of 5 trials employed cluster design [21-25], whereas the remaining studies used a parallel group design. The majority of studies (51.8%) were conducted in nurses [23,24,26-37] followed by pharmacists (14.8%) [38-41], mental health therapists (11.1%) [21,25,42], dentists (7.4%) [43,44], midwives [22], physical therapists [45], patient care personnel [46], and substance abuse counselors [47]. The evaluated interventions included blended learning [35]; CD-ROM and emails [43]; computer-assisted instruction (CAI), computer-based training, or computer-mediated training [22,23,26,28,29,34,36,38, 41,42,44-46]; CD-ROM [24,30-33,37,47]; PowerPoint presentation [39,40,46]; and software [21,25,27]. The duration of the intervention ranged from 50 min [28,38] to 3 months [21,25,31,46]. The intensity ranged from 15 min [44] to 2.4 h [37]. Comparison groups included no intervention [22,26,32,33,37,43-45,47], blended learning [21,25], and traditional learning [23,24,27-31,34-36,38-42,46]. Primary outcomes included knowledge in 20 studies [21-24,26-48], skills in 9 studies [22,24,31,35,37,38,41-43], attitudes in 7 studies [26,30,35,40,41,44,45], satisfaction in 9 studies [23,25,28, 33,35,36,38,40,42], and patient-centered outcomes in 2 studies [22,25].

### Risk of Bias in Included Studies

**Figure 2:**
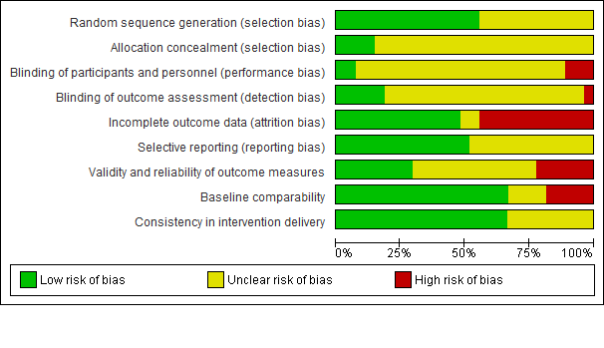
Risk of bias graph: review authors' judgements about each risk of bias item presented as percentages across all included studies.

We present our judgments about each risk of bias item for all included studies as (summary) percentages in Figure 2.

**Figure 3:**
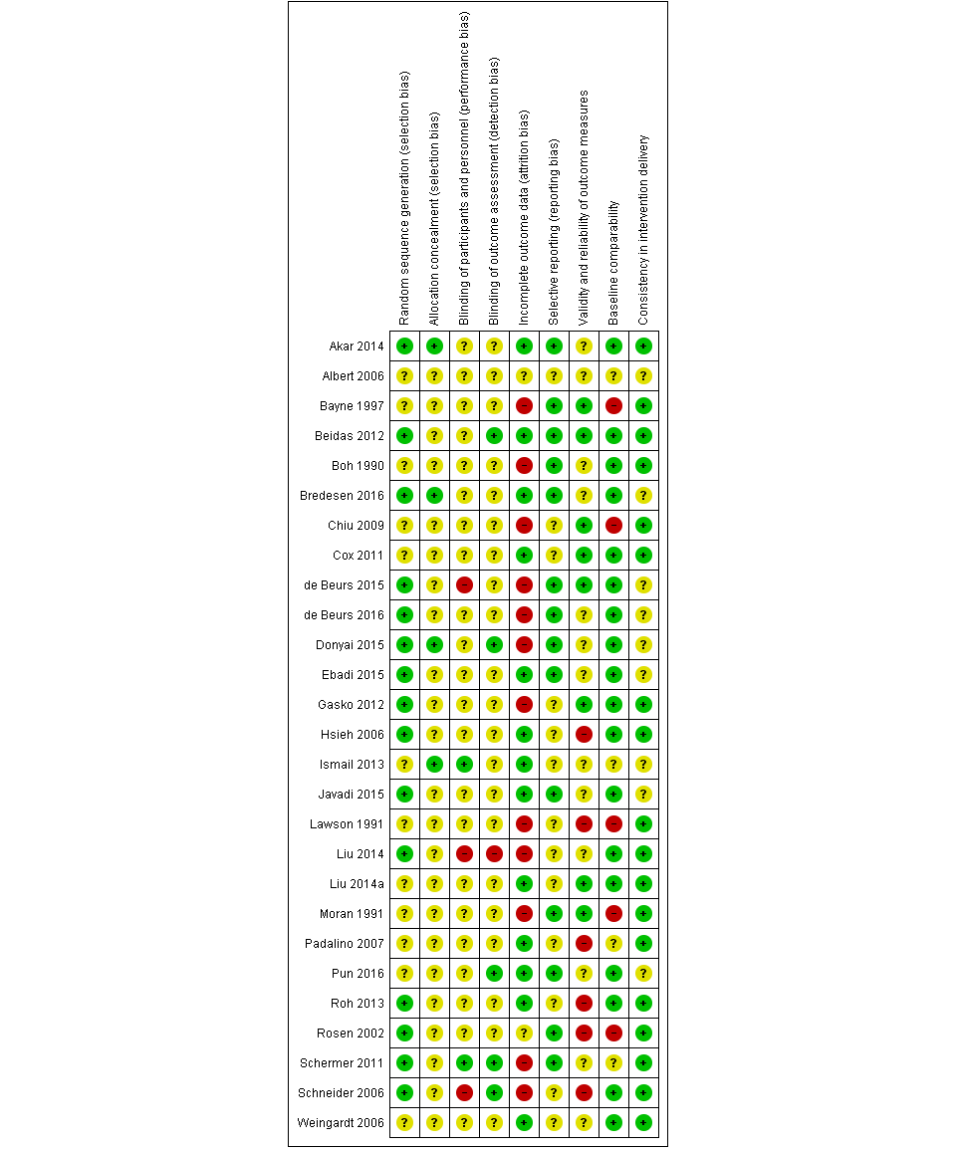
Risk of bias summary: review authors' judgements about each risk of bias item for each included study.

Figure 3 shows separate judgments about each risk of bias item for each included study.

The risk of bias was predominantly low for random sequence generation (55.5% of the studies), selective reporting, baseline comparability, and consistency in intervention delivery. The risk of bias was predominantly unclear for allocation concealment blinding of participants, personnel, or outcome assessors. A total of 12 studies (44.4%) had a high risk of attrition bias; 6 studies (22.2%) had a high risk of bias for validity and reliability of outcome measures; and 5 studies (18.5%) had a high risk of bias for baseline comparability. In total, 3 studies (11.1%) had a high risk of performance bias; and 1 study (3.7%) had a high risk of detection bias. For cRCTs, all 5 studies had a low risk of bias for selective recruitment of cluster participants.

**Table 1:** Characteristics of included studies

Author (year), reference, country	Population/health profession (N)	Field of study/condition/health problem	Intervention type	Control	Outcomes (measurement instrument)	Results (continuous or dichotomous)
Akar (2014) [46], Turkey	Patient care personnel (96)	Testicular cancer	PowerPoint presentation	T^a^	Knowledge (MCQ 26-items)	Mean (SD) 12.0 (1.9) vs 10.4 (3.7); *P*=.005
Albert (2006) [43], United States	Dentists (184)	Tobacco addiction	CD-ROM and email	NL^b^	1. Skills	1. *P*<.01
					2. Knowledge	2. *P*<.05
Bayne (1997) [26], United States	Nurses (67)	Drug overdose	CAI^c^	NL	1. Knowledge (test 20-items)	1. Mean (SD) 82.1 (11.88) vs 81.1 (13.0)
					2. Satisfaction	2. —^d^
					3. Attitude (Q^e^)	3. —
Beidas (2012) [42], United States	Mental health therapist (115)	Anxious children	CBL^f^	T	1. Skills (checklist)	1. Mean (SD) 17.4 (1.81) vs 17.4 (1.83)
					2. Knowledge (test 20-items)	2. Mean (SD) 3.6 (1.47) vs 4.1 (1.45)
					3. Satisfaction (Q)	3. Mean (SD) 50.8 (5.9) vs 53.7 (5.4); (*P*<.001)

Boh (1990) [38], United States	Pharmacists (105)	Osteoarthritis	Computer-based simulation	T	1. Knowledge (MCQ^g^ 25-items)	1. Mean (SD) 76.0 (8.59) vs 65.73 (9.65); (*P*<.005)
					2. Skills (simulation)	2. Mean (SD) 32.9 (8.15) vs 26.5 (10.90)
					3. Satisfaction (Q)	3. —
Bredesen (2016) [27], Norway	Nurses (44)	Pressure ulcer prevention	Software	T	1. Knowledge (a. Braden scale and b. pressure ulcer classification)	a. NS^h^
						b. Fleiss kappa=0.20 (0.18-0.22) vs 0.27 (0.25-0.29)
Chiu (2009) [28], Taiwan	Nurses (84)	Stroke	CAI	T	1. Knowledge (Q 15-items)	1. Mean (SD) 34.7 (2.4) vs 33.7 (5.0); (*P*=.21)
					2. Satisfaction (Q 16-items)	2. Mean (SD) 61.5 (8.40) vs 60.3 (7.80); (*P*=.51)
Cox (2009) [29], United States	Nurses (60)	Pressure ulcers	CBL	T	Knowledge (Single choice questionnaire)	Mean (SD) 90.3 (4.9) vs 92.9 (3.3); (*P*=.717)
de Beurs (2015) [21], Holland	Psychiatric departments (567^i^)	Suicide prevention	Software (train-the-trainer)^a^	BL^j^	Knowledge (Q 15-items)	Mean (SD) 26.6 (3.1) vs 24.1 (2.3)
de Beurs 2016 [46], Holland	Psychiatric departments (881^i^)	Suicide prevention	Software (train-the-trainer)^k^	BL	1. Patient-centered outcome (Beck scale 19-items)	1. Mean (SD) 4.2 (13.4) vs 4.9 (10.5)
					2. Satisfaction (4-point scale)	2. Mean (SD) 6.8 (4.4) vs 6.8 (4.3)
Donyai (2015) [39], United Kingdom	Pharmacy professional (48)	Continuing professional development case scenarios	PowerPoint presentation	T	Knowledge (score)	Mean difference 9.9; 95% CI 0.4 to 19.3; (*P*=.04)
Ebadi (2015) [30], Iran	Nurses (90)	Biological incidents	CD-ROM	T	1. Knowledge (MCQ 34-items)	1. Mean (SD)=24.3 (5.1) vs 13.9 (3.2) (*P*<.001)
					2. Attitude (visual analogue scale 0-100)	2. Mean (SD) 81.59 (15.21) vs 54.4 (20.24); (*P*<.001)
Gasko (2012) [31], United States	Nurse anesthetists (29)	Regional anesthesia	CD-ROM	T	Skills (Q 16 criteria)	Mean (SD) 33 (7) vs 35 (10); (*P*<.05)
Hsieh (2006) [44], United States	Dentists (174)	Domestic violence	CBL	NL	1. Knowledge (Q 24-items)	1. (*P*<.01)
					2. Attitude	2. (*P*<.01)
Ismail (2013) [22], United Kingdom	Midwives (25)	Perineal trauma	CBL	NL	Patient-centered outcomes	Delta=0.7%; 95% CI −10.1 to 11.4; (*P*=.89)
Javadi (2015) [40], Iran	Pharmacists (71)	Contraception and sexual dysfunctions	PowerPoint presentation	T	1. Knowledge (MCQ 23-items)	1. Mean (SD) 68.46 (16.60) vs 50.75 (17.58); (*P*<.001)
					2. Satisfaction (Q 5-items)	2. —
					3. Attitude (scale 14-items)	3. Median 28 vs 27; (*P*=.18)
Lawson (1991) [41], United States	Pharmacists (50)	Financial management	CBL	T	1. Skills (Q 25-items)	1. Mean (SD) 15.63 (3.37) vs 16.04 (3.35)
					2. Attitude	2. (*P*=.082)
Liu (2014) [32], Taiwan	Psychiatric nurses (216)	Case management	CD-ROM	NL	Knowledge (MCQ 20-items)	Delta= 0.37; 95% CI –3.3 to 4.0; (*P*=.84)
Liu (2014) [33], Taiwan	Nursing personnel (40)	Nursing care management	CD-ROM	NL	Knowledge (Q)	Mean (SD) 91 (8.6) vs 58 (20.4)
Moran (1991) [45], United States	Physical therapists (41)	Wound care	CAI	NL	1. Knowledge (test 13-items)	1. Mean (SD) 10.85(1.56) vs 9.05(1.77); (*P*<.004)
					2. Attitude (survey)	2. —
Padalino (2007) [34], Brazil	Nurses (49)	Quality training program	CBL	T	Knowledge (Q)	Mean (SD) 19.4(1.7) vs 17.8(3.2); (*P*=.072)
Pun (2016) [35], Hong Kong	Nurses (40)	Hemodialysis management	BL	T	1. Knowledge (MCQ and fill-in-the blank questions)	1. Mean (SD) 24 (1.03) vs 17.45 (2.74); (*P*<.001)
					2. Skills (checklist 39-items)	2. Mean (SD) 149.3 (19.42) vs 113.65 (21.23); (*P*<.001)
					3. Attitude (3-item checklist 7-point Likert scale)	3. Mean (SD) 1.83 (0.03)^l^
					4. Satisfaction (7-point Likert scale)	4. Range 2.10 to 2.75 (0.55 to 0.94)^I^
Roh (2013) [36], Korea	Nurses (38)	Advanced life support	CBL	T	Satisfaction (Q 20-items)	Mean (SD) 7.64 (1.04) vs 7.43 (1.34); (*P*=.588)
Rosen (2002) [23], United States	Nurses (173)	Mental health and aging	CBL	T	1. Knowledge (test)	1. Mean (SD) 90.0 (9.1) vs 84.0 (11.2); (*P*=.004)
					2. Satisfaction (Q)	2. (*P*<.0001)
Schermer (2011) [24], Holland	Nurses (1135)	Spirometry	CD-ROM	T	Skills (test)	OR^m^ 1.2, 95% CI 0.6 to 2.5; (*P*=.663)
Schneider (2006) [37], United States	Nurses (30)	Medication administration	CD-ROM	NL	Skills (observation)	OR 0.38, 95% CI 0.19 to 0.74; (*P*=.004)
Weingardt (2006) [47], United States	Substance abuse counselor (166)	Substance abuse	CD-ROM	NL	Knowledge (MCQ)	(*P*<.01)

^a^T: traditional.

^b^NL: no learning.

^c^CAI: computer-assisted instruction.

^d^—: not reported.

^e^Q: questionnaire.

^f^CBL: computer-based learning.

^g^MCQ: multiple choice questionnaire.

^h^NS: not significant.

^i^Total number of patients or professions.

^j^BL: blended learning.

^k^Intervention also included blended learning (Web-based plus traditional learning).

^l^Data for the intervention group only.

^m^OR: odds ratio.

### Effects of Interventions

#### Offline Digital Education (CD-ROM) Versus No Intervention or Traditional Learning

##### Primary Outcomes

###### Knowledge

**Figure 4:**
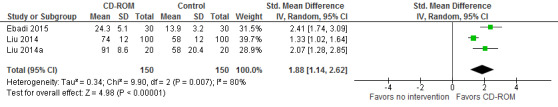
Forest plot of comparison: Offline digital education (CD-ROM) versus no intervention, outcome: Knowledge.

A meta-analysis of 3 studies [30,32,33] considered to be homogeneous enough found that compared with no intervention, offline digital education (CD-ROM) may increase knowledge in nurses (SMD 1.88; 95% CI 1.14 to 2.62; low certainty evidence, Figure 4). There was a substantial level of heterogeneity of the pooled studies (Tau^2^=.34; χ^2^=9.90; *P*=.007; *I^2^*=80%; low certainty evidence).

A total of 2 studies did not report sufficient data that could be included in the meta-analysis. Weingardt [47] reported that compared with no intervention, CD-ROM probably improves substance abuse counselors’ knowledge (*P*<.01; moderate certainty evidence). Albert [43] reported that compared with no intervention, CD-ROM and email may slightly improve dentists’ knowledge (*P*<.05; low certainty evidence).

###### Skills

Schneider [37] reported an increase in nurses’ skills (decreased core 1 error rates) between baseline and postintervention periods in the intervention group (OR 0.38, 95% CI 0.19 to 0.74; *P*=.004; low certainty evidence). Albert [43] reported that compared with no intervention, the offline digital education (CD-ROM and email) intervention may slightly improve dentists’ skills (*P*<.01; low certainty evidence). Gasko [31] reported that the CD-ROM intervention may have little or no effect on nurse anesthetists’ skills compared with traditional learning (mean 33 [SD 7] vs mean 35 [SD 10]; low certainty evidence). Schermer [24] reported that compared with traditional training (joint baseline workshop), CD-ROM may slightly improve the rate of adequate tests (32.9% vs 29.8%; OR 1.2, 95% CI 0.6 to 2.5; *P*=.663; low certainty evidence).

###### Satisfaction

Liu [33] reported that 87% of participants in the CD-ROM groups agreed or strongly agreed that the program was flexible (mean 4.28; low certainty evidence). There was no comparison group for this outcome. For a summary of the effects of these comparisons on knowledge, skills, and satisfaction, see SoF in Multimedia Appendix 4.

#### Offline Digital Education (Computer-Assisted Training) Versus No Intervention or Traditional Learning

##### Primary Outcomes

###### Knowledge

**Figure 5:**
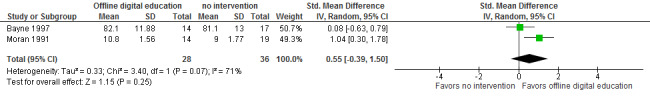
Forest plot of comparison: Offline digital education (computer-assisted training) versus no intervention, outcome: Knowledge.

A meta-analysis of 2 studies [26,45] considered to be homogeneous enough found that compared with no intervention, the effects of offline digital education (computer-assisted training [CAT]) on nurses and physical therapists’ knowledge were uncertain (SMD 0.55; 95% CI –0.39 to 1.50; very low certainty evidence; Figure 5).

A substantial level of heterogeneity of the pooled studies was detected (Tau^2^=.33; χ^2^=3.40; *P*=.07; *I^2^*=71%). One study [44] did not present data that could be included in the meta-analysis for this outcome. Hsieh reported that compared with no intervention, offline digital education may improve dentists’ knowledge (*P*<.01; low certainty evidence).

Beidas [42] reported that compared with routine training, offline digital education (computer training) may have little or no effect on community mental health therapists’ knowledge postintervention (mean 17.45 [SD 1.83] vs mean 17.42 [SD 1.81]; *P*=.26; low certainty evidence). Boh [38] found that compared with traditional learning, an intervention (audio cassette and microcomputer simulation) may improve pharmacists’ knowledge postintervention (mean 65.7 [SD 9.6] vs mean 76 [SD 8.5]; *P*<.005; low certainty evidence). Chiu [28] reported that compared with traditional training, offline digital education (CAI) may slightly improve nurses’ knowledge at 4 weeks (mean 33.7 [SD 5.0] vs mean 34.7 [SD 2.4]; *P*=.21; low certainty evidence). Cox [29] found that compared with traditional training, offline digital education (computer-based learning) may have little or no effect on nurses’ knowledge postintervention (mean 92.9 [SD 3.3] vs mean 90.3 [SD 4.9]; *P*=.717; low certainty evidence). Padalino [34] reported that compared with traditional classroom training, offline digital education (computer-mediated training) may slightly improve nurses’ knowledge postintervention (mean 17.8 [SD 3.2] vs mean 19.4 [SD 1.7]; *P*=.072; low certainty evidence). Rosen [23] found that compared with usual education, offline digital education (computer-based training) may improve nurses’ knowledge at 6 months (mean 84 [SD 11.2] vs mean 90 [SD 9.1]; *P*=.004; low certainty evidence). Taken together, these results suggest that computer-assisted interventions may slightly improve various health professionals’ knowledge, but the quality of evidence was low and results were mixed.

###### Skills

**Figure 6:**
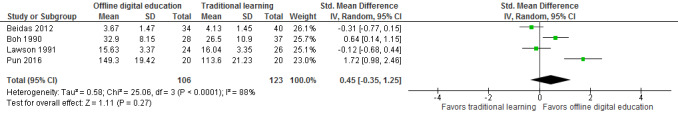
Forest plot of comparison: Offline digital education (computer-assisted training) versus traditional learning, outcome: Skills.

A meta-analysis of 4 studies [35,38,41,42] found that compared with traditional training, the effects of CAT on skills in community (mental health) therapists, nurses, and pharmacists were uncertain (SMD 0.45; 95% CI –0.35 to 1.25; very low certainty evidence; Figure 6). Heterogeneity of the pooled studies was considerable (Tau^2^=.58; χ^2^=25.06; *P*<.0001; *I^2^*=88%).

###### Attitudes

In Moran [45], 93% of respondents reported a strong agreement or an agreement with the statement that computer-assisted instructions were helpful. There was no comparison group for this outcome (low certainty evidence). Hsieh [44] reported that compared with no intervention, the computer-based tutorial group may improve dentists’ attitudes (*P*<.01; low certainty evidence). Lawson [41] found that compared with traditional education, offline digital education may have little or no effect on participants’ attitudes concerning expected helpfulness (*P*=.082; low certainty evidence).

###### Satisfaction

**Figure 7:**
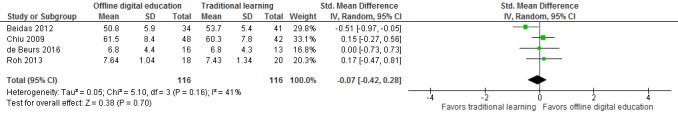
Forest plot of comparison: Offline digital education (computer-assisted training) versus traditional learning, outcome: Satisfaction.

A meta-analysis of 4 studies [25,28,36,42] considered to be homogeneous enough found that compared with traditional training, offline digital education may have little effect or no difference on satisfaction scores in nurses and mental health therapists (SMD –0.07; 95% CI –0.42 to 0.28; low certainty evidence; Figure 7). A moderate level of heterogeneity of the pooled studies was detected (Tau^2^=.05; χ^2^=.10; *P*=.16; *I^2^*=41%).

A total of 2 studies [23,38] were not included in the meta-analysis for this outcome as they did not report a sufficient amount of data for pooling. Boh [38] found that compared with traditional learning, offline digital education (audio cassette and microcomputer simulation) may have little or no effect on pharmacists’ satisfaction postintervention (low certainty evidence). Rosen [23] found that compared with usual education, offline digital education (computer-based training) may improve nurses’ satisfaction at 6 months (*P*<.0001; low certainty evidence).

###### Patient-Centered Outcomes

Ismail [22] reported that compared with no intervention, offline digital education may have little or no effect on the average percentage of women reporting perineal pain on sitting and walking at 10 to 12 days (mean difference [MD]=0.7%; 95% CI −10.1 to 11.4; *P*=.89; low certainty evidence).

##### Secondary Outcomes

Only 1 study [27] mentioned the costs of offline digital education. Bayne and Bindler [27] reported the costs as US $54 per participant in the computer-assisted group compared with US $23 per participant in the no intervention control group. For a summary of the effects of these comparisons on all outcomes, see SoF Multimedia Appendix 4.

#### Offline Digital Education (Software, PowerPoint) Versus Blended Learning or Traditional Learning

##### Primary Outcomes

###### Knowledge

**Figure 8:**
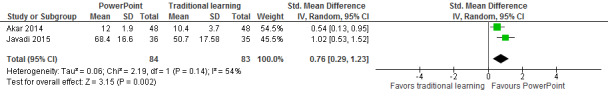
Forest plot of comparison: Offline digital education (PowerPoint) versus traditional learning, outcome: Knowledge.

A meta-analysis of 2 studies [40,46] considered to be homogeneous enough found that compared with traditional learning, a PowerPoint presentation may improve the knowledge of patient care personnel and pharmacists (SMD 0.76; 95% CI 0.29 to 1.23; low certainty evidence; Figure 8). A considerable level of heterogeneity of the pooled studies was detected (Tau^2^=.06; χ^2^=2.19; *P*=.14; *I*^2^=54%).

One study did not report sufficient data to be included in the meta-analysis. Donyai [39] reported that compared with traditional learning, a PowerPoint presentation may improve pharmacy professionals’ knowledge (MD=9.9; 95% CI 0.4 to 19.3; *P*=.04).

de Beurs [21] reported that compared with blended learning, offline digital education (software) may improve mental health professionals’ knowledge (mean [SD] 26.6 (3.1) vs 24.1 (2.3); *P*<.001; low certainty evidence).

###### Satisfaction

de Beurs [25] reported that compared with blended learning, offline digital education (software) may have little effect or no difference on patients’ satisfaction at 3 months (mean [SD] 6.8 (4.4) vs 6.8 (4.3); low certainty evidence).

###### Patient-Centered Outcomes

de Beurs [25] reported that compared with blended learning, offline digital education (software) may have little effect or no difference on patients’ suicidal ideation at 3 months (mean [SD] 4.2 (13.4) vs 4.9 (10.5); low certainty evidence). For a summary of the effects of these comparisons on all outcomes, see SoF Multimedia Appendix 4.

There was not enough data included in any of the pooled analyses to allow sensitivity analyses to be conducted. Similarly, given the small number of trials contributing data to outcomes within different comparisons in this review, a formal assessment of potential publication bias was not feasible.

## Discussion

We summarized and critically evaluated evidence for effectiveness of offline digital education for improving knowledge, skills, attitudes, satisfaction, and patient-centered outcomes in postgraduate health professions except medical doctors. A total of 27 studies with 4618 participants met the eligibility criteria. We found highly diverse studies in different professions and evidence to support the effectiveness of certain types of offline digital education such as CD-ROM and PowerPoint compared with no intervention or traditional learning in improving learners’ knowledge. For other outcomes (and comparators), the evidence was less compelling in improving learners’ skills, attitudes, satisfaction, and patient-related outcomes.

### Overall Completeness and Applicability of Evidence

We identified 4 studies from upper middle-income countries (Brazil, Iran, and Turkey), and the remaining studies were conducted in high-income countries (Hong Kong, Korea, the Netherlands, Norway, the United Kingdom, and the United States). Only 4 studies (15%) were conducted during the 1990s and the remaining studies were from 2000 onward. In 15 studies (55.5%), information about the frequency of the interventions was missing, thereby often making it difficult to analyze in depth and interpret the findings. Similarly, economic evaluations of the interventions were missing in 26 (96%) studies.

### Quality of the Evidence

Overall, the quality of evidence was low or very low. We assessed the quality of evidence using the Grading of Recommendations, Assessment, Development, and Evaluations system and presented the findings in SoF Multimedia Appendix 4 for all comparisons. The reasons for downgrading the evidence most commonly pertained to the high risk of bias. For instance, only 13 (48.1%) of the studies reported complete outcome data. Reducing the dropout rate might reduce the risk of attrition bias and further improve the quality of the studies. Only 8 studies (29.6%) had a low risk of bias for validity and reliability of outcome measures. This issue of nonvalidated measurement tools has repeatedly been raised and is paramount to advance the field [49]. Only 2 studies (7.4%) adequately described blinding of participants and personnel. As with many educational interventions, blinding of participants or personnel might prove challenging. However, we highlighted the need for more adequate descriptions of masking to further reduce the risk of performance bias and allow clearer judgments to be made. We also downgraded the overall quality of evidence for inconsistency (where there was a high level of heterogeneity, ie, *I*^2^>50%). Overall, there was a moderate-to-considerable level of heterogeneity of meta-analyses (*I*^2^ range 41% to 88%); and 4 (out of 5) meta-analyses had *I*^2^>50%. More reasons for downgrading included indirectness (we downgraded once for 1 outcome only—where there were differences in the population used). Participants were not homogeneous and ranged from nurses, pharmacists, mental health therapists, dentists, midwives and obstetricians, physical therapists, patient care personnel to substance abuse counselors. Other sources of indirectness also stemmed from heterogeneous interventions (their duration, frequency, and intensity), comparison groups, and outcome assessment tools ranging from multiple choice or single choice questionnaires, tests, observations, checklists, scales, surveys, visual analogue scales, and simulations. Finally, we also downgraded for imprecision where the sample size was small. The included studies also failed to provide details of sample size and power calculations and may have therefore been underpowered and unable to detect change in learning outcomes.

### Strengths and Limitations of the Review

This systematic review has several important strengths that include comprehensive searches without any language limitations, robust screening, data extraction and risk of bias assessments, and a critical appraisal of the evidence. However, some limitations must be acknowledged while interpreting the results of this study. First, we considered subgroup analyses to be unfeasible because of the insufficient number of studies under the respective outcomes and professional groups. However, we minimized potential biases in the review process and maintained its internal validity by strictly adhering to the guidelines outlined by Higgins et al [18].

### Agreements and Disagreements With Other Studies or Reviews

A review by Al-Jewair [50] found some evidence to support the effectiveness of computer-assisted learning in improving knowledge gains in undergraduate or postgraduate orthodontic students’ or orthodontic educators’ knowledge, but no definite conclusions were reached; and future research was recommended. Rosenberg [51] concluded that computer-aided learning is as effective as other methods of teaching and can be used as an adjunct to traditional education or as a means of self-instruction of dental students. Based on 4 mixed-results RCTs, Rosenberg [52] was unable to reach any conclusions on knowledge gains and recommended more high-quality trials evaluating the effectiveness of computer-aided learning in orthodontics. However, we are familiar with newer technologies being currently evaluated for the same outcomes; MOOCs or mLearning can play a very important role in health professions education such as improving clinical knowledge and promoting lifelong learning [53-54]. We are also aware of recent reviews, which reached similar conclusions [55-62]. For example, digital education seems to be at least as effective (and sometimes more effective) as traditional education in improving dermatology, diabetes management, or smoking cessation–related skills and knowledge [58,61,62]. Most of these reviews, however, stressed the inconclusiveness of overall findings mainly because of the low certainty of the evidence.

### Conclusions

Offline digital education may potentially play a role in the education of health professionals, especially in LMICs, where there is a lack of access to Web-based digital education for a variety of reasons, including cost; and there is some evidence to support the effectiveness of these interventions in improving the knowledge of health professionals. However, because of the existing gaps in the evidence base, including limited evidence for other outcomes; lack of subgroup analyses, for example, CD-ROM or PowerPoint; low and very low quality of the evidence, the overall findings are inconclusive. More research especially evaluating patient-centered outcomes, costs, and safety (adverse effects); involving those subgroups; and originating from LMICs is needed. Such research should be adequately powered, be underpinned by learning theories, use valid and reliable outcome measures, and blind outcome assessors.

## Appendix

Multimedia Appendix 1 MEDLINE (Ovid) search strategy.

Multimedia Appendix 2 Characteristics of excluded studies.

Multimedia Appendix 3 Results of the included studies.

Multimedia Appendix 4 Summary of findings tables.

## Abbreviations

CAI: computer-assisted instruction
CAT: computer-assisted training
cRCT: cluster randomized controlled trial
LMIC: low- and middle-income country
MD: mean difference
mLearning: mobile learning
MOOC: massive open online course
OR: odds ratio
RCT: randomized controlled trial
SMD: standardized mean difference
SoF: Summary of Findings
