# Correction: Offline Digital Education for Postregistration Health Professions: Systematic Review and Meta-Analysis by the Digital Health Education Collaboration

**DOI:** 10.2196/20316

**Published:** 2020-06-23

**Authors:** Pawel Posadzki, Malgorzata M Bala, Bhone Myint Kyaw, Monika Semwal, Ushashree Divakar, Magdalena Koperny, Agnieszka Sliwka, Josip Car

**Affiliations:** 1 Centre for Population Health Sciences Lee Kong Chian School of Medicine Nanyang Technological University Singapore Singapore Singapore; 2 Chair of Epidemiology and Preventive Medicine Department of Hygiene and Dietetics Jagiellonian University Medical College Krakow Poland; 3 Family Medicine and Primary Care Lee Kong Chian School of Medicine Nanyang Technological University Singapore Singapore Singapore; 4 Province Sanitary-Epidemiological Station of Lesser Poland Public Health and Health Promotion Department Krakow Poland; 5 Institute of Physiotherapy, Faculty of Health Sciences Jagiellonian University Medical College Krakow Poland

The authors of “Offline Digital Education for Postregistration Health Professions: Systematic Review and Meta-Analysis by the Digital Health Education Collaboration” (J Med Internet Res 2019;21(4):e12968) noticed an error in one of their author affiliations.

The affiliation for Malgorzata M Bala has been changed from:

2Health Services Department, The Agency for Health Technology Assessment and Tariff System, Warsaw, Poland

to:

2Chair of Epidemiology and Preventive Medicine, Department of Hygiene and Dietetics, Jagiellonian University Medical College, Krakow, Poland

The correction will appear in the online version of the paper on the JMIR website on June 23, 2020, together with the publication of this correction notice. Because this was made after submission to PubMed, PubMed Central, and other full-text repositories, the corrected article has also been resubmitted to those repositories.

