# Supplementary material for: Offline Digital Education for Postregistration Health Professions: Systematic Review and Meta-Analysis by the Digital Health Education Collaboration
Source: J Med Internet Res. 2019 Apr 24;21(4):e12968. doi: 10.2196/12968 (PMC6505374; doi:10.2196/12968)
Supplement: Multimedia Appendix 2 [file jmir_v21i4e12968_app2.docx]

**Appendix 3**

**Characteristics of excluded studies**

***Benharash 2012***

| **Reason for exclusion** | Inappropriate population |
| --- | --- |

***Browne 2004***

| **Reason for exclusion** | Inappropriate design |
| --- | --- |

***Danley 2004***

| **Reason for exclusion** | Inappropriate population |
| --- | --- |

***Devitt 1998***

| **Reason for exclusion** | Inappropriate population |
| --- | --- |

***Dimeff 2011***

| **Reason for exclusion** | Inappropriate population |
| --- | --- |

***Friedman 1999***

| **Reason for exclusion** | Inappropriate population |
| --- | --- |

***Horn 1992***

| **Reason for exclusion** | Inappropriate population |
| --- | --- |

***Kay 2001***

| **Reason for exclusion** | Inappropriate comparator |
| --- | --- |

***Lange 1997***

| **Reason for exclusion** | Inappropriate design |
| --- | --- |

***Leopold 2005***

| **Reason for exclusion** | Inappropriate population |
| --- | --- |

***Lieberman 2002***

| **Reason for exclusion** | Inappropriate population |
| --- | --- |

***Maunder 2010***

| **Reason for exclusion** | Inappropriate comparator |
| --- | --- |

***Perkins 2010***

| **Reason for exclusion** | Inappropriate population |
| --- | --- |

***Schell 2004***

| **Reason for exclusion** | Inappropriate population |
| --- | --- |

***Sholomskas 2006***

| **Reason for exclusion** | Inappropriate population |
| --- | --- |

***Stout 2012***

| **Reason for exclusion** | Inappropriate population |
| --- | --- |
