# Supplementary material for: Offline Digital Education for Postregistration Health Professions: Systematic Review and Meta-Analysis by the Digital Health Education Collaboration
Source: J Med Internet Res. 2019 Apr 24;21(4):e12968. doi: 10.2196/12968 (PMC6505374; doi:10.2196/12968)
Supplement: Multimedia Appendix 3 [file jmir_v21i4e12968_app3.docx]

Multimedia Appendix 3: Results of the included studies

| **Author (year), reference, Country** | **Population/ health profession (N)** | **Field of study/** **condition/health problem** | **Intervention** | | | | **Control** | **Outcomes (MI)** | **Results (continuous or dichotomous)** |
| --- | --- | --- | --- | --- | --- | --- | --- | --- | --- |
|  |  |  | **Intervention type** | **Duration** | **Frequency** | **Intensity** |  |  |  |
| Akar (2014) [21],  Turkey | Patient care personnel (96) | Testicular cancer | PowerPoint® presentation | 12 weeks | - | 45 minutes | T | Knowledge (MCQ 26-items) | Mean (SD)=12.0 (1.9) vs 10.4 (3.7) p=.005 |
| Albert (2006) [22], USA | Dentists (184) | Tobacco addiction | CD-ROM and email | - | - | - | NL | 1.Skills 2.Knowledge | 1.p<.01  2.p<.05 |
| Bayne (1997) [23],  USA | Nurses (67) | Drug overdose | CAI | 3 hours | Once | 3 hours | NL | 1.Knowledge (Test 20-items)  2.Satisfaction  3.Attitude (Q) | 1.Mean (SD)=82.1 (11.88) vs  81.1 (13.0)  2.-  3.- |
| Beidas (2012) [24], USA | Mental health therapist (115) | Anxious children | CBL | 6 hours | Once | 6 hours | T | 1.Skills (Checklist) 2.Knowledge (Test 20-items)  3.Satisfaction (Q) | 1.Mean (SD)=17.4 (1.81) vs 17.4 (1.83)  2.Mean (SD)=3.6 (1.47) vs 4.1 (1.45)  3. Mean (SD) = 50.8 (5.9) vs 53.7 (5.4) (p<.001) |
| Boh (1990) [25], USA | Pharmacists (105) | Osteoarthritis | CBS | 50 minutes | Twice | 2 hours | T | 1.Knowledge (MCQ 25-items)  2.Skills (Simulation) 3.Satisfaction (Q) | 1.Mean (SD)=76.0 (8.59) vs 65.73 (9.65) (p<.005)  2.Mean (SD)=32.9 (8.15) vs 26.5 (10.90)  3.- |
| Bredesen (2016)  [26], NOR | Nurses (44) | Pressure ulcer prevention | Software | - | - | - | T | 1.Knowledge (a. Braden Scale and b. pressure ulcer classification) | a. n.s.  b. kappa Fleiss = 0.20 (0.18–0.22) vs 0.27 (0.25–0.29) |
| Chiu (2009) [27],  Taiwan | Nurses (84) | Stroke | CAI | 50 minutes | - | - | T | 1.Knowledge (Q 15-items) 2.Satisfaction (Q 16-items) | 1.Mean (SD)=34.7 (2.4) vs 33.7 (5.0) (p=.21)  2.Mean (SD) = 61.5 (8.40) vs 60.3 (7.80) (p=.51) |
| Cox (2009) [28],  USA | Nurses (60) | Pressure ulcers | CBL | 2 weeks | - | 6 programmes | T | Knowledge (SCQ) | Mean (SD)=90.3 (4.9) vs 92.9 (3.3) (p=.717) |
| de Beurs (2015) [29], NL | Psychiatric departments (567##) | Suicide prevention | Software (Train-the-Trainer^)a^ | 3 months | - | 1 hour | BL | Knowledge (Q 15-items) | Mean (SD)=26.6 (3.1) vs 24.1 (2.3) |
| de Beurs 2016 [21], NL | Psychiatric departments (881##) | Suicide prevention | Software (Train-the-Trainer)^a^ | 3 months | - | 1 day | BL | 1. Patient-centered outcome (Beck Scale 19-items)  2.Satisfaction (4-point scale) | 1.Mean (SD)=4.2 (13.4) vs 4.9 (10.5)  2.Mean (SD)=6.8 (4.4) vs 6.8 (4.3) |
| Donyai (2015) [30], UK | Pharmacy professional (48) | CPD case scenarios | PowerPoint® presentation | - | - | - | T | Knowledge (Score) | MD=9.9; 95 % CI 0.4–19.3; (p=.04) |
| Ebadi (2015) [31], Iran | Nurses (90) | Biological incidents | CD-ROM | 2 weeks | - | - | T | 1.Knowledge (MCQ 34-items)  2.Attitude (VAS 0-100) | 1.Mean (SD) = 24.3 (5.1) vs 13.9 (3.2) (p<.001)  2. Mean (SD) = 81.59 (15.21) vs 54.4 (20.24) (p<.001) |
| Gasko (2012) [32],  USA | Nurse anesthetists (29) | Regional anesthesia | CD-ROM | 3 months | Twice | 20 minutes | T | Skills (Q 16 criteria) | Mean (SD) = 33 (7) vs 35 (10) (p<.05) |
| Hsieh (2006) [33],  USA | Dentists (174) | Domestic violence | CBL | - | Once | 15 minutes | NL | 1.Knowledge (Q 24-items)  2.Attitude | 1. (p<.01)  2. (p<.01) |
| Ismail (2013) [34],  UK | Midwives (25) | Perineal trauma | CBL | 2 hours | Once | - | NL | Patient-centered outcomes | Δ=0.7%; 95% CI −10.1% to 11.4% (p=.89) |
| Javadi (2015) [35], Iran | Pharmacists (71) | Contraception and sexual dysfunctions | PowerPoint® presentation | 1 day | Twice | 1.5 hours | T | 1.Knowledge (MCQ 23-items)  2.Satisfaction (Q 5-items)  3.Attitude (Scale 14-items) | 1. Mean (SD)=68.46 (16.60) vs 50.75 (17.58) (p<.001)  2.-  3.Median = 28 vs 27 (p=.18) |
| Lawson (1991) [36],  USA | Pharmacists (50) | Financial management | CBL | 2 hours | Once | 2 hours | T | 1.Skills (Q 25-items)  2.Attitude | 1.Mean (SD) =15.63 (3.37) vs 16.04 (3.35)  2.(p=.082) |
| Liu (2014) [37],  Taiwan | Psychiatric nurses (216) | Case management | CD-ROM | 1 hours | - | 1 hours | NL | Knowledge (MCQ 20-items) | Δ = 0.37; 95% CI -3.3 to 4.0 (p=.84) |
| Liu (2014) [38],  Taiwan | Nursing personnel (40) | Nursing care management | CD-ROM | - | - | 5 units | NL | Knowledge (Q) | Mean (SD)=91 (8.6) vs 58 (20.4) |
| Moran (1991) [39],  USA | Physical therapists (41) | Wound care | CAI | 2 weeks | - | - | NL | 1.Knowledge (test 13-items)  2.Attitude (survey) | 1.Mean (SD) = 10.85(1.56) vs 9.05(1.77) (p<.004)  2.- |
| Padalino (2007) [40],  Brazil | Nurses (49) | Quality training program | CBL | 2 hours | Once | 40 minutes | T | Knowledge (Q) | Mean (SD)=19.4(1.7) vs 17.8(3.2) (p=.072) |
| Pun (2016) [41],  Hong Kong | Nurses (40) | Hemodialysis management | BL | 2 weeks | Twice | 15 minutes | T | 1.Knowledge (MCQ and fill-in-the blank questions)  2.Skills (checklist 39-items)  3.Attitude (three-item checklist-7 point Likert scale) 4.Satisfaction (7-point Likert scale) | 1.Mean (SD)=24 (1.03) vs 17.45 (2.74) (p<.001)  2.Mean (SD) = 149.3 (19.42) vs 113.65 (21.23) (p<.001)  3.Mean (SD) =1.83 (0.03)^b^  4.range = 2.10 to 2.75 (0.55 to 0.94)# |
| Roh (2013) [42],  Korea | Nurses (38) | Advanced life support | CBL | 2 hours | Once | 2 hours | T | Satisfaction (Q 20-items) | Mean (SD)=7.64 (1.04) vs 7.43 (1.34) (p=.588) |
| Rosen (2002) [43],  USA | Nurses (173) | Mental health and aging | CBL | - | - | 35-45 minutes | T | 1.Knowledge (Test)  2.Satisfaction (Q) | 1.Mean (SD) = 90.0 (9.1) vs 84.0 (11.2) (p=.004)  2. (p<.0001) |
| Schermer (2011) [44],  NL | Nurses (1135) | Spirometry | CD-ROM | 3 hours | - | 30 minutes | T | Skills (Test) | OR=1.2; 95% CI 0.6 to 2.5 (p=.663) |
| Schneider (2006) [45],  USA | Nurses (30) | Medication administration | CD-ROM | 2 weeks | - | 2.4 hours | NL | Skills (Observation) | OR=0.38; 95% CI 0.19 to 0.74 (p=.004) |
| Weingardt (2006) [46],  USA | Substance abuse counsellor (166) | Substance abuse | CD-ROM | 90 minutes | Once | 60 minutes | NL | Knowledge (MCQ) | (p<.01) |
