# Supplementary material for: Offline Digital Education for Postregistration Health Professions: Systematic Review and Meta-Analysis by the Digital Health Education Collaboration
Source: J Med Internet Res. 2019 Apr 24;21(4):e12968. doi: 10.2196/12968 (PMC6505374; doi:10.2196/12968)
Supplement: Multimedia Appendix 4 [file jmir_v21i4e12968_app4.docx]

## Multimedia Appendix 4: Summary of findings tables

# Summary of findings table 1: Effects of offline digital education (CD-ROM) on knowledge, skills and satisfaction

| **Offline digital education (CD-ROM) versus no intervention or traditional learning** | | | | | | |
| --- | --- | --- | --- | --- | --- | --- |
| **Patient or population:** post-registration health professionals (dentists, nurses, psychiatric nurses, substance abuse counsellors) **Setting:** various settings **Intervention(s):** offline digital education (CD-ROM)  **Comparison:** no intervention or traditional learning | | | | | | |
| **Outcomes** | **Anticipated absolute effects^z^ (95% CI)** | | **Relative effect (95% CI)** | **№ of participants (studies)** | **Quality of the evidence (GRADE)** | **Comments** |
|  | **Risk with no intervention** | **Risk with Digital Education (CD-ROM)** |  |  |  |  |
| **Knowledge** Assessed with: MCQ and Questionnaire | The mean knowledge was 43.3 | The mean knowledge in the intervention group was 1.88 standard deviations more (1.14 more to 2.62 more) | Not estimable | 300 (3 RCTs) | ⊕⊕⊝⊝ LOW ^a, b^ | Weingardt (2006) (166 participants) found that digital education (CD-ROM) probably improves slightly substance abuse counsellors' knowledge (moderate certainty^c^).  Albert (2006) (184 participants) found that digital education (CD-ROM and email) may improve slightly dentists' knowledge (low certainty^d^). |
| **Skills**  Assessed with: observation | See comment | See comment | Not estimable | See comment | See comment | Schneider (2006) (30 participants) reported an increase in nurses' skills (decreased core 1 error rates) between baseline and post intervention periods in the offline digital education (CD-ROM) group (low certainty^d^). Albert (2006) reported that compared with no intervention, the offline digital education (CD-ROM and email) intervention may improve slightly dentists' skills (low certainty evidence^d^). Gasko (2012) reported that the CD-ROM intervention may have little or no effect on nurse anaesthetists' skills compared with traditional learning (low certainty evidence^d^). Schermer (2011) reported that compared with traditional training (joint baseline workshop), CD-ROM may improve slightly the rate of adequate tests was (low certainty evidence^d^). |
| **Satisfaction**  Assessed with: 5-point Likert like scale | See comment | See comment | Not estimable | See comment | See comment | Liu (2014) (216 participants) reported that 87% of participants in the offline digital education (CD-ROM) intervention agreed or strongly agreed that the program was flexible (low certainty^d^). There was no comparison group for this outcome. |
| ^z^**The risk in the intervention group** (and its 95% confidence interval) is based on the assumed risk in the comparison group and the **relative effect** of the intervention (and its 95% CI).  **CD-ROM:** Compact Disc Read-Only Memory; **MCQ:** Multiple choice questionnaire. | | | | | | |
| **GRADE Working Group grades of evidence** **Low quality:** Our confidence in the effect estimate is limited: The true effect may be substantially different from the estimate of the effect | | | | | | |

^a^ High risk of performance, detection and attrition bias (-1).

^b^ Heterogeneity was considerable *I^2^* 80% (-1).

^c^ Downgraded as single study contributing to results (-1).

^d^ Downgraded on risk of bias (-1); Single study contributing to results (-1).

**Summary of findings table 2**: Effects of offline digital education (computer assisted learning) on knowledge, skills, attitudes, satisfaction, patient centered outcomes and economic outcomes

| **Offline digital education (computer assisted training) vs no intervention or traditional learning** | | | | | | |
| --- | --- | --- | --- | --- | --- | --- |
| **Patient or population:** post-registration health professionals; patients (women with perineal trauma)  **Setting:** various settings **Intervention:** Offline digital education (CAT) **Comparison:** no intervention or traditional learning | | | | | | |
| **Outcomes** | **Anticipated absolute effects^z^ (95% CI)** | | **Relative effect (95% CI)** | **№ of participants (studies)** | **Quality of the evidence (GRADE)** | **Comments** |
|  | **Risk with no intervention** | **Risk with digital education (CAT)** |  |  |  |  |
| **Knowledge**  Assessed with: Tests | The mean knowledge was 0 | The mean knowledge in the intervention group was 0.55 standard deviations more (0.39 fewer to 1.5 more) | Not estimable | 64 (2 RCTs) | ⊕⊝⊝⊝ VERY LOW ^a, b, d^ | *Offline digital education vs no intervention*  Hsieh (2006) (174 participants) reported that offline digital education may improve dentists' knowledge (low certainty^e^).  *Offline digital education vs traditional learning*  Beidas (2012) (115 participants) reported that offline digital education may have little or no effect on community mental health therapists' knowledge post intervention (low certainty^f^). Boh (1990) (105 participants) found that offline digital education may improve pharmacists' knowledge post intervention (low certainty^e^). Chiu (2009) (84 participants) reported that the intervention may improve slightly nurses' knowledge at 4 weeks (low certainty^f^). Cox (2011) (60 participants) found that the intervention may have little or no effect on nurses' knowledge post intervention (low certainty^g^). Padalino (2007) (49 participants) reported that the intervention may improve slightly nurses' knowledge post intervention (low certainty^h^). Rosen (2002) (3 clusters) found that the intervention probably improves nurses' knowledge at 6 months (moderate certainty^i^). |
| **Skills**  Assessed with:  Test; Simulation and Questionnaire | The mean skills was 40 | The mean skills in the intervention group was 0.45 standard deviations more (0.35 fewer to 1.25 more) | Not estimable | 229 (4 RCTs) | ⊕⊝⊝⊝ VERY LOW^j, k, l^ | - |
| **Attitudes**  Assessed with:  Seven-point Likert scale (4 items) | See comment | See comment | Not estimable | See comment | See comment | *Offline digital education vs no intervention*  Hsieh (2006) reported that offline digital education may improve dentists' attitudes (low certainty^5^).  *Offline digital education vs traditional education*  Lawson (1991) found that offline digital education may have little or no effect on participants' attitudes concerning expected helpfulness (low certainty evidence^e^). |
| **Satisfaction**  Assessed with: Questionnaires | The mean satisfaction was 32 | The mean satisfaction in the intervention group was 0.07 standard deviations fewer (0.42 fewer to 0.28 more) | Not estimable | 232 (4 RCTs) | ⊕⊕⊝⊝ LOW ^a, c^ | Boh (1990) (105 participants) found that compared with traditional learning, offline digital education may have little or no effect on pharmacists' satisfaction post intervention (low certainty^5^). Rosen (2002) found that compared with traditional education, offline digital education may improve nurses' satisfaction at 6 months (low certainty^5^). |
| **Patient centered outcomes**  Assessed with:  Four-item scale ranging from ‘none’ to ‘severe’ | See comment | See comment | Not estimable | See comment | See comment | Ismail (2013) (25 participants) reported that offline digital education may have little or no effect on the average percentage of women reporting perineal pain when sitting and walking at 10 to 12 days when compared with no intervention (low certainty^e^). |
| **Economic outcomes** | See comment | See comment | Not estimable | See comment | See comment | Bayne (1997) reported the costs of $54/participant in the offline digital education compared with $23/participant in the no intervention group (low certainty^e^). |
| ^z^**The risk in the intervention group** (and its 95% confidence interval) is based on the assumed risk in the comparison group and the **relative effect** of the intervention (and its 95% CI). **CAT:** computer assisted training. | | | | | | |
| **GRADE Working Group grades of evidence** **Low quality:** Our confidence in the effect estimate is limited: The true effect may be substantially different from the estimate of the effect **Very low quality:** We have very little confidence in the effect estimate: The true effect is likely to be substantially different from the estimate of effect | | | | | | |

###### Footnotes

^a^ Downgraded on risk of bias (-1).

^b^ Downgraded on inconsistency- heterogeneity was high *I*^2^ >70% (-1).

^c^ Downgraded on indirectness - heterogeneous populations (-1).

^d^ Downgraded on imprecision small sample (-1).

^e^ Downgraded on risk of bias (-1); Single study contributing to results (-1).

^f^ Downgraded as single study contributing to results (-1); Imprecision-small sample size (-1).

^g^ Downgraded on imprecision-small sample size (-1); Downgraded on inconsistency as effects are in the opposite directions (-1).

^h^ Downgraded on imprecision-small sample size (-1); Downgraded on risk of bias for validity and reliability of outcome measures (-1).

^i^ Downgraded on risk of bias for validity and reliability of outcome measures and baseline comparability (-1).

^j^ Downgraded on risk of bias (-1) two studies Boh (1990); Lawson (1991) were judged as high on attrition bias; Lawson (1991) also scored high on validity and reliability of outcome measures; and baseline comparability (-1).

^k^ Downgraded as effects are in different directions (-1).

^l^ Downgraded as small sample size and wide confidence intervals (-1).

## Summary of findings table 3: Effects of Offline digital education (software, PowerPoint) on knowledge, satisfaction and patient centered outcomes

| **Offline digital education vs traditional or blended learning** | | | | | | |
| --- | --- | --- | --- | --- | --- | --- |
| **Patient or population:** post-registration health professionals **Setting:** various settings **Intervention:** offline digital education (CAT) **Comparison:** traditional or blended learning | | | | | | |
| **Outcomes** | **Anticipated absolute effects^z^ (95% CI)** | | **Relative effect (95% CI)** | **№ of participants (studies)** | **Quality of the evidence (GRADE)** | **Comments** |
|  | **Risk with classroom based or conventional learning** | **Risk with offline digital education** |  |  |  |  |
| **Knowledge**  Assessed with: Scores, Questionnaires,  MCQ | The mean skills was 30.5 | The mean knowledge in the intervention group was 0.76 standard deviations higher (0.29 higher to 1.23 higher) | Not estimable | 167 (2 RCTs) | ⊕⊕⊝⊝ LOW ^a, b^ | *Offline digital education (PowerPoint) vs traditional learning*  Donyai (2015) reported that compared with traditional learning, PowerPoint Presentation may improve pharmacy professionals’ knowledge (low certainty evidence^2^).  *Offline digital education (software) versus blended learning*  de Beurs (2015) reported that compared with blended learning, offline digital education (software) may improve mental health professionals’ knowledge (low certainty evidence^2^). |
| **Satisfaction**  Assessed with: Scale | See comment | See comment | Not estimable | See comment | See comment | de Beurs (2016) reported that compared with blended learning, offline digital education (software) may have little effect or no difference on patients’ satisfaction at 3 months (low certainty evidence^2^). |
| **Patient centered outcomes**  Assessed with:  Beck Sale | See comment | See comment | Not estimable | See comment | See comment | de Beurs (2016) reported that compared with blended learning, offline digital education (software) may have little effect or no difference on patients’ suicidal ideation at 3 months (low certainty evidence^2^). |
| **Adverse outcome** | See comment | See comment | Not estimable | See comment | See comment | No studies reported adverse events. |
| ^z^**The risk in the intervention group** (and its 95% confidence interval) is based on the assumed risk in the comparison group and the **relative effect** of the intervention (and its 95% CI). **CAT:** computer assisted training; **MCQ:** Multiple choice questionnaire. | | | | | | |
| **GRADE Working Group grades of evidence** **High quality:** We are very confident that the true effect lies close to that of the estimate of the effect **Moderate quality:** We are moderately confident in the effect estimate: The true effect is likely to be close to the estimate of the effect, but there is a possibility that it is substantially different **Low quality:** Our confidence in the effect estimate is limited: The true effect may be substantially different from the estimate of the effect **Very low quality:** We have very little confidence in the effect estimate: The true effect is likely to be substantially different from the estimate of effect | | | | | | |

###### Footnotes

^a^ Downgraded on risk of bias (-1); and on inconsistency- heterogeneity was high *I*^2^ >50% (-1).

^b^ Downgraded on risk of bias (-1); single study contributing to results (-1).
